# Effect of epidural spinal cord stimulation on female sexual function after spinal cord injury

**DOI:** 10.3389/fnins.2023.1155796

**Published:** 2023-04-05

**Authors:** Claire Shackleton, Soshi Samejima, Tiev Miller, Rahul Sachdeva, Ann Parr, Uzma Samadani, Theoden Netoff, Shea Hocaloski, Stacy Elliott, Matthias Walter, David Darrow, Andrei Krassioukov

**Affiliations:** ^1^International Collaboration on Repair Discoveries, Faculty of Medicine, University of British Columbia, Vancouver, BC, Canada; ^2^Division of Physical Medicine and Rehabilitation, Department of Medicine, University of British Columbia, Vancouver, BC, Canada; ^3^Department of Neurosurgery, University of Minnesota, Minneapolis, MN, United States; ^4^Department of Bioinformatics and Computational Biology, University of Minnesota, Minneapolis, MN, United States; ^5^Minneapolis Veterans Affairs Medical Center, Minneapolis, MN, United States; ^6^Department of Biomedical Engineering, University of Minnesota, Minneapolis, MN, United States; ^7^GF Strong Rehabilitation Centre, Vancouver Coastal Health, Vancouver, BC, Canada; ^8^Department of Psychiatry, Vancouver Coastal Health Authority, Vancouver, BC, Canada; ^9^Department of Urologic Sciences, Vancouver Coastal Health Authority, Vancouver, BC, Canada; ^10^Department of Urology, University Hospital Basel, University of Basel, Basel, Switzerland; ^11^Hennepin County Medical Center, Minneapolis, MN, United States

**Keywords:** spinal cord injuries, neuromodulation, sexual dysfunction, sexual health, sexuality, sexual arousal, orgasm

## Abstract

Sexual dysfunction is a common consequence for women with spinal cord injury (SCI); however, current treatments are ineffective, especially in the under-prioritized population of women with SCI. This case-series, a secondary analysis of the Epidural Stimulation After Neurologic Damage (E-STAND) clinical trial aimed to investigate the effect of epidural spinal cord stimulation (ESCS) on sexual function and distress in women with SCI. Three females, with chronic, thoracic, sensorimotor complete SCI received daily (24 h/day) tonic ESCS for 13 months. Questionnaires, including the Female Sexual Function Index (FSFI) and Female Sexual Distress Scale (FSDS) were collected monthly. There was a 3.2-point (13.2%) mean increase in total FSFI from baseline (24.5 ± 4.1) to post-intervention (27.8 ± 6.6), with a 4.8–50% improvement in the sub-domains of desire, arousal, orgasm and satisfaction. Sexual distress was reduced by 55%, with a mean decrease of 12 points (55.4%) from baseline (21.7 ± 17.2) to post-intervention (9.7 ± 10.8). There was a clinically meaningful change of 14 points in the International Standards for Neurological Classification of Spinal Cord Injury total sensory score from baseline (102 ± 10.5) to post-intervention (116 ± 17.4), without aggravating dyspareunia. ESCS is a promising treatment for sexual dysfunction and distress in women with severe SCI. Developing therapeutic interventions for sexual function is one of the most meaningful recovery targets for people with SCI. Additional large-scale investigations are needed to understand the long-term safety and feasibility of ESCS as a viable therapy for sexual dysfunction.

**Clinical Trial Registration:**https://clinicaltrials.gov/ct2/show/NCT03026816, NCT03026816.

## Introduction

Female sexual dysfunction is a common and etiologically multifactorial complication of spinal cord injury (SCI; Anderson et al., 2007; Krassioukov and Elliott, 2017). Disruption of sensorimotor and autonomic control following SCI drastically affects physiological responses during sexual activity, including decreased genital sensation, vaginal lubrication, orgasmic capacity, and increased dyspareunia (Krassioukov and Elliott, 2017; Bel HajYoussef et al., 2018). Low self-confidence, self-esteem, and poor body image are common consequences of SCI in women, which together with physiological changes, can reduce sexual desire and satisfaction (Elliott et al., 2017). Secondary consequences of SCI [e.g. autonomic dysreflexia, neurogenic bowel (NBD) and lower urinary tract (LUT) dysfunction (NLUTD)] further compromise a woman’s sexual desire and activity (Elliott et al., 2017). These sexual concerns are not considered dysfunctions unless they are associated with psychological distress (Faubion and Rullo, 2015). Despite the complexity of sexual dysfunction following SCI, women remain sexually active. In fact, women rate sexual function as a key priority for quality of life following SCI (Anderson, 2004).

Despite relatively promising treatments for men with SCI, there are limited treatment options and a paucity of research for female sexual dysfunction following SCI (Courtois and Charvier, 2015; Elliott et al., 2017). Previous reports highlight that female sexual needs after SCI are underprioritized (Courtois and Charvier, 2015; Krassioukov and Elliott, 2017; Thrussell et al., 2018). The majority of women with SCI have expressed dissatisfaction with the quality and quantity of sexuality-related rehabilitation services, and lack of treatments (Elliott et al., 2017; Krassioukov and Elliott, 2017). Fundamental changes to sensation and genital vasocongestion secondary to nerve injury, to date, have not been successfully addressed in the neurogenic population (Krassioukov and Elliott, 2017).

Recently, there has been a surge of case reports demonstrating that spinal cord stimulation (SCS) may ameliorate crucial autonomic functions, including LUT and bowel function (Walter et al., 2018; DiMarco et al., 2021). Considering the proximity of the neural pathways that modulate LUT/bowel function with those that modulate sexual function, neurostimulation may too restore neural control for genital arousal and orgasm in individuals with SCI. To date, only two case reports have shown anecdotal evidence of epidural SCS (ESCS) for improving sexual responses in a male (Harkema et al., 2011) and a female with SCI (Darrow et al., 2019). The National Institute of Biomedical Imaging and Bioengineering called a consortium of multiple stakeholders to discuss the key issues related to SCS for individuals with SCI (Pettigrew et al., 2017). Sexual function was identified as one of the top research priorities and it was hypothesized that ESCS would improve sexual arousal and sexual satisfaction for individuals with SCI (Pettigrew et al., 2017). In this report, we present improvement in perceived sexual function in women with SCI who participated in the Epidural Stimulation After Neurologic Damage (E-STAND) trial.

## Materials and methods

This preliminary case-series is a secondary analysis of sexual function in females with SCI who underwent the E-STAND clinical trial in the United States (ClinicalTrials.gov number: NCT03026816; Darrow et al., 2019; Peña Pino et al., 2020; Darrow et al., 2022). The clinical trial was approved by the local Institutional Review Board, the Food and Drug Administration Investigational Device Exemption, and was conducted in accordance with the Declaration of Helsinki. This study followed the “Strengthening the Reporting of Observational Studies in Epidemiology (STROBE) Statement” according to the EQUATOR guidelines for reporting observational studies (Cuschieri, 2019).

### Participant characteristics

Participants with chronic (>1 year since injury), traumatic SCI were recruited, from August 2017, if they met the following criteria: greater than 22 years of age, sensorimotor complete classification [American Spinal Injury Association Impairment Scale (AIS)] A or B with a neurological level of injury between cervical (C)6 and thoracic (T)10, full arm and hand strength, and intact segmental reflexes below the level of injury. Participants were excluded if they had medical or psychological comorbidities that would significantly increase the risk of operation, severe dysautonomia with systolic blood pressure fluctuation below 50 or above 200 mmHg, contractures, pressure ulcers, recurrent urinary tract infection refractory to antibiotics, unhealed spinal fracture, botulinum toxin injections in the previous 6 months, or were pregnant. Active recruitment for the E-STAND clinical trial is ongoing, with a suspected end date of January 2027. All participants provided written informed consent and adhered to the study protocol.

### Stimulation settings and study assessments

**Figure 1:**
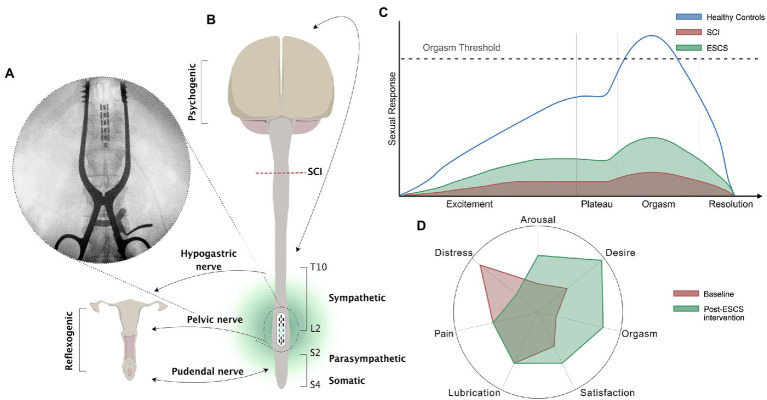
Schematic of the neural control of female sexual function, the female sexual response cycle, and the effects of SCS. **(A)** X-ray of the thoracolumbar spinal column of participant 02 with chronic T4, AIS A, SCI during implantation of ESCS. The epidural electrode was implanted at T11-T12 vertebral level; **(B)** Schematic diagram showing neural control of sexual responses in females with SCI above T10. Neurological pathways convey sexual responses in women through the pudendal nerve (S2-S4 somatosensory) innervating the clitoris and external labia, the pelvic nerve (S2-S4 parasympathetic) innervating the vagina and cervix, and the hypogastric nerve (T10-L2 sympathetic) innervating the cervix and uterus. Reflexogenic arousal refers to vaginal lubrication that occurs as a result of sensual touching and genital stimulation. Psychogenic arousal refers to vaginal lubrication that occurs as a result of arousal in the brain (e.g., through hearing, seeing, feeling, or fantasy); **(C)** The four-phase (excitement, plateau, orgasm, and resolution) linear model of the human sexual response adapted from Masters and Johnson (1966). The schematic presents the normal human sexual response (blue line) and the proposed depressed sexual response in women after SCI (red shading). The beneficial effect of ESCS on the four phases of the sexual response cycle in women with SCI is demonstrated by the green shading. Although criticized, this physiological response curve is based on 10,000 recordings of able-bodied participants, and gives us great insight into how physiological human sexual responses may be generated after SCI, allowing for possible therapeutic interventions; **(D)** A radial plot demonstrating the six domains of the Female Sexual Function Index, and sexual distress according to the Female Sexual Distress Scale, in three females from the E-STAND trial at baseline (red shading) and post ESCS intervention (green shading).

Three female participants were implanted with a 16-contact paddle lead epidural stimulator at T11-T12 (Tripole and Proclaim Elite, Abbott, Plano, TX, United States; Figure 1A). Participants were provided with a programmer and after a month of gradual adjustment to time and amplitude of stimulation, they were allowed to utilize specific stimulation settings for different goals, such as volitional movement, spasticity control, core strength, and autonomic functions. As the primary outcomes of the ESTAND trial were motor and cardiovascular benefits, the implanted stimulators were switched on (i.e., active) up to 24 h per day to derive the necessary benefits during daily activity. A detailed description of study methods and ESCS parameters can be found in previous publications (Darrow et al., 2019; Peña Pino et al., 2020; Darrow et al., 2022). Neurological function was collected pre- and post-intervention using the International Standards for Neurological Classification of Spinal Cord Injury (ISNCSCI; Kirshblum et al., 2011) exam, and self-reported questionnaires on LUT [i.e., Neurogenic Bladder Symptom Score (NBSS)] (Welk et al., 2018) and bowel function [i.e., NBD Score (NBDS)] (Krogh et al., 2006) were completed monthly. Validated female-specific questionnaires [i.e., Female Sexual Distress Scale (FSDS)] (Derogatis et al., 2002, 2008) and [Female Sexual Function Index (FSFI)] (Rosen et al., 2000; Meston, 2003; Wiegel et al., 2005; Meston et al., 2020) were completed prior to implantation, and each subsequent month for 13 months of ESCS, 30–45 days apart.

### Statistical approach

In order to overcome the inherent limitation of an open-label study, in which there is no control/Sham group, each participant served as their own control using a pre-post design. The current study used a single-subject design with data presented as means ± standard deviation (SD). Due to the primary efficacy end-point of the larger ESTAND trial (phase II pre-post clinical trial), our secondary analysis of sexual function reports pre and post data only.

Percentage change was calculated to contextualize the magnitude of the differences from pre- to post-intervention. When possible, comparisons with available data, such as minimal clinically important differences, are provided.

## Results

### Participant characteristics

Three female individuals, aged (mean ± SD) 47.7 ± 11.2 years, with chronic (11.7 ± 3.1 years), T4–T8 SCI participated in E-STAND clinical trial. Two participants were married, and one was in a long-term partnership at the time of the trial. Baseline sensorimotor evaluations using the ISNCSCI exam showed all participants had sensorimotor complete injuries (AIS A). All participants presented with NLUTD (i.e., NBSS total score 15.3 ± 11.4) and minor to severe NBD (i.e., NBDS 12 ± 3.6) at baseline. A summary of participant characteristics, sensorimotor, LUT and bowel functions is provided in Table 1. Baseline evaluations of sexual function using the FSFI (24.5 ± 4.1) and the FSDS (21.7 ± 17.2) indicated all participants experienced sexual dysfunction and distress, respectively. Participant sexual function domain and monthly scores (Supplementary Tables S1, S2), and a list of medications (Supplementary Table S3) are provided in the Supplementary Appendix.

**Table 1:** Participant characteristics at baseline and post-intervention.

	Demographics												
Participant	Age (years)			Time since injury (years)^†^		NLI		AIS					
01	56			15		T8		A					
02	35			11		T4		A					
03	52			9		T5		A					
*N* = 3*	47.7 ± 11.2			11.7 ± 3.1		T4–T8		A					
	**International standards for neurological classification of spinal cord injury**												
	**Motor function**					**Sensory function**				**Anal sensorimotor function**			
	**UEMS total (0–50)**			**LEMS total (0–50)**		**LT total (0–112)**		**PP total (0–112)**		**Voluntary anal contraction**		**Deep anal pressure**	
	**Pre**		**Post**	**Pre**	**Post**	**Pre**	**Post**	**Pre**	**Post**	**Pre**	**Post**	**Pre**	**Post**
01	50		50	0	0	58	68	56	68	No	No	No	No
02	50		50	0	0	40	54	58	54	No	No	No	No
03	50		50	0	0	48	52	46	52	No	No	No	No
*N* = 3*	50.0 ± 0.0		50.0 ± 0.0	0.0 ± 0.0	0.0 ± 0.0	48.7 ± 9.0	58.0 ± 8.7	53.3 ± 6.4	58.0 ± 8.7	No	No	No	No
Δ (%)	0			0		19.2		8.8					
	**Neurogenic bladder symptom score**									**Neurogenic Bowel Dysfunction Score**			
	**Incontinence (0–29)**		**Storage/voiding (0–22)**		**Consequence (0–23)**		**Quality of life (0–4)**			**Overall score (0–47)**		**Dysfunction severity**	
	**Pre**	**Post**	**Pre**	**Post**	**Pre**	**Post**	**Pre**		**Post**	**Pre**	**Post**	**Pre**	**Post**
01	14	13	9	8	4	5	1		2	9	9	Minor	Minor
02	0	0	1	3	4	6	1		1	16	14	Severe	Severe
03	7	0	3	3	0	0	2		1	11	9	Moderate	Minor
*N* = 3*	7.0 ± 7.0	4.3 ± 7.5	4.3 ± 4.2	4.7 ± 2.9	2.7 ± 2.3	3.7 ± 3.2	1.3 ± 0.6		1.3 ± 0.6	12.0 ± 3.6	10.7 ± 2.9		
Δ (%)	−38.1		7.7		38.5		0			−11.1			

^†^Indicates the number of years post injury at the time of surgical implantation. *Mean ± SD, Δ (%) = Percent change from baseline to post-intervention. AIS, American Spinal Injury Association Impairment Scale; LEMS, Lower Extremity Motor Score; NLI, Neurological Level of Injury; LT, Light Touch; PP, Pin Prick; UEMS, Upper Extremity Motor Score.

### Sexual function and distress

**Figure 2:**
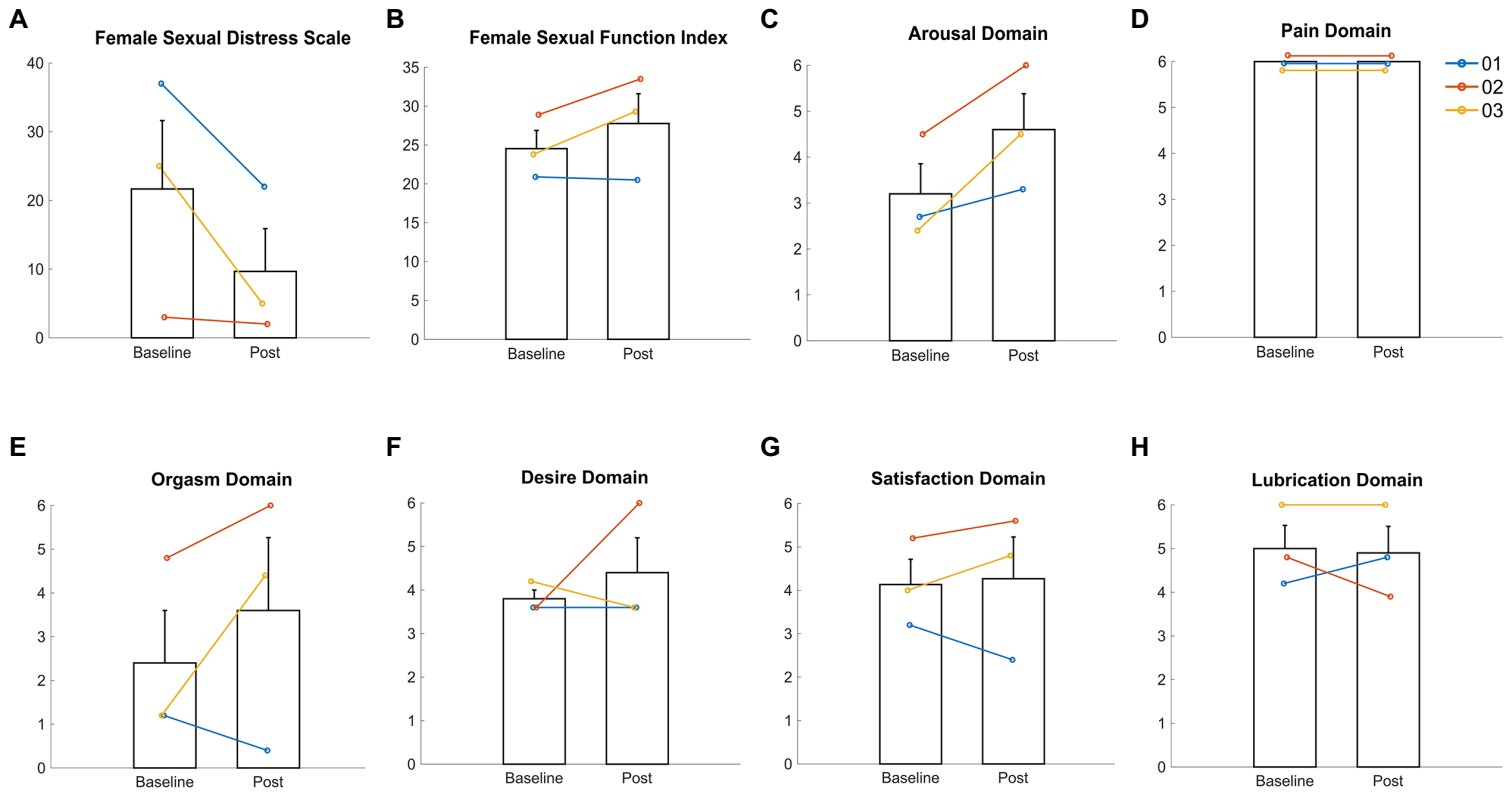
Female sexual distress and sexual function in three female participants with thoracic sensorimotor complete SCI. **(A)** Female sexual distress measured by the Female Sexual Distress Scale; **(B)** Total sexual function measured by the Female Sexual Function Index (FSFI); **(C)** Arousal domain of the FSFI; **(D)** Pain domain of the FSFI; **(E)** Orgasm domain of the FSFI; **(F)** Desire domain of the FSFI; **(G)** Satisfaction domain of the FSFI; **(H)** Lubrication domain at FSFI; Bar plots represent the mean scores at baseline and post-intervention; Errors bars represent the standard deviation; Individual participant responses presented by coloured lines: 01 (blue); 02 (red); 03 (yellow).

There was a 3.2-point (13.2%) mean increase in overall FSFI from baseline (24.5 ± 4.1) to 13-month post-intervention (27.8 ± 6.6; Figure 2B). However, the ESCS-dependent improvement in FSFI was observed in two participants (P 02 and 03), with a 18.9% increase in overall sexual function from baseline (26.4 ± 3.6) to post-intervention (31.4 ± 2.9), while the third participant (P 01) experienced no change in overall FSFI function from baseline (20.9 ± 1.6) to post-intervention (20.5 ± 1.9). Although there was a general upwards trend in sexual function scores over time, results indicate that there was individual variation between monthly time points and participants (Supplementary Table S2).

For all participants, there was a mean increase in scores across the FSFI sub-domains (Figures 2C,E–G) ranging from 0.2–1.4 points (4.8–50%) from baseline to post-intervention (desire: 3.8 ± 0.3 vs. 4.4 ± 1.4; arousal: 3.2 ± 1.1 vs. 4.6 ± 1.4; orgasm: 2.4 ± 2.1 vs. 3.6 ± 2.9; satisfaction: 4.1 ± 1.0 vs. 4.3 ± 1.7). Only lubrication showed a mean decrease of 0.1 point (2%) from baseline (5.0 ± 0.9) to post-intervention (4.9 ± 1.1; Figure 2H). Participants did not report any dyspareunia at baseline (6.0 ± 0.0), during, or after the intervention (6.0 ± 0.0; Figure 2D).

A reduction in sexual distress (Figure 2A) was observed across all participants, with a mean decrease of 12 points (55.4%) from baseline (21.7 ± 17.2) to post-intervention (9.7 ± 10.8).

### Sensorimotor function and urinary/bowel incontinence

According to previously established minimal clinically important difference (MCID = 5.19 points) for total sensory scores (Scivoletto et al., 2013), there was a clinically meaningful change of 14 points in sensory function (i.e., total sensory score) from baseline (102 ± 10.5) to post-intervention (116 ± 17.4; Table 1). All participants had full upper extremity motor function (UEMS: 50/50) and no lower extremity motor function (LEMS: 0/50) at baseline, with no change post-intervention. The incontinence domain of the NBSS improved from baseline (7.0 ± 7.0) to post-intervention (4.3 ± 7.5) and there was a small improvement in overall NBDS from baseline (12.0 ± 3.6) to post-intervention (10.7 ± 2.9; Table 1).

## Discussion

Despite a lack of treatment options for female sexual dysfunction following SCI, this study provides novel findings demonstrating the possibility of using ESCS to reduce sexual dysfunction and distress in women with chronic sensorimotor complete SCI, without adverse effects.

Two of the three participants had improved FSFI scores after the intervention, suggesting that ESCS can elicit small but clinically meaningful changes in sexual dysfunction according to an established cut-off for diagnosing female sexual dysfunction (i.e., FSFI ≤26.55; Figure 2; Wiegel et al., 2005). It is likely that ESCS activated and modulated the somatic [Sacral (S)2-S4], sympathetic [S2-S4] and parasympathetic [T10- lumbar (L)2] circuits (Figure 1) involved in genital arousal and sexual function. However, notably, ESCS was not specifically targeted to restoration of sexual function, and yet, participants still experienced improvement in several sexual function domains.

Women with SCI are significantly less likely to achieve orgasm and take considerably longer to achieve orgasm than their able-bodied counterparts, particularly in those without an intact sacral reflex arc (Figure 1C; Courtois et al., 2017; Krassioukov and Elliott, 2017). However, the female participants in our study, with sensorimotor complete thoracic injuries, reported improved scores in the orgasm domain of the FSFI following the SCS intervention (Figure 1D). Even if a physiological orgasm is not experienced or reported by the participants, the sexual experience can be completed by psychological satisfaction associated with the emotionally rewarding and pleasant sexual experience (Kreuter et al., 2008). Furthermore, sexual stimuli can result in nonsexual rewards, such as partner intimacy and emotional well-being, which can drive and enhance sexual willingness and satisfaction. Satisfaction is reportedly lower in women after SCI, with at least 25% experiencing diminished satisfaction with their sexual life (Kreuter et al., 2008). However, the participants in our study reported improved sexual satisfaction with ESCS (Figure 1D). Additionally, sexual desire and arousal domains were also positively influenced by ESCS (Figure 1D). In this study, improvements in self-reported outcomes for one FSFI domain (i.e., arousal) appeared to in turn influence another (e.g., orgasm, satisfaction). The potential overlap in specific domains (i.e., arousal, desire) has been acknowledged previously (Wiegel et al., 2005; Meston et al., 2020) and may be of particular importance in neurogenic populations.

In this study, subjective awareness of lubrication did not show any improvement after ESCS, however, this may be a consequence of vaginal transudate requiring estrogenized vaginal epithelial cells and that two of the participants were over the age of 50 (Krassioukov and Elliott, 2017). Alternatively, ESCS may not be sufficient to induce the autonomic physiological changes required for improved vaginal lubrication.

Previous evidence shows that relative to able-bodied controls, women with SCI report significantly higher sexual distress.^23^ Participants in this study reported a substantial decrease in sexual distress from pre- to post-intervention (Figure 1D). The 50% reduction in sexual distress after ESCS represents a clinically meaningful change based on an established cut-off for diagnosing female sexual distress (i.e., FSDS ≥11; Derogatis et al., 2008). In particular, women with SCI are more prone to experiencing anorgasmia than their able-bodied counterparts (Figure 1C; Courtois et al., 2017; Bel HajYoussef et al., 2018), which can lead to significant distress. Our participants had improved orgasm domain scores, possibly reducing sexual distress. Similarly, improvements in sensory scores during the intervention may also have contributed to reduced sexual distress. Importantly, although ESCS improved sensory function, it had no negative impact on dyspareunia. One of the most commonly reported and distressing sexual complications after SCI is urinary and bowel incontinence (Anderson et al., 2007). Therefore, aside from improvements in multiple aspects of sexual function, the reduction in urinary and bowel incontinence may too have positively impacted the reduced sexual distress.

## Conclusion

Despite considerable variance in demographics, injury-related and personal factors which impact female sexuality, we show that ESCS could potentially offer treatment for sexual dysfunction and distress in women with severe SCI. ESCS reduced overall sexual dysfunction, with benefits in desire, arousal, orgasm, satisfaction, and distress. Although the small sample size is restrictive for statistical power and limits the generalizability of these findings to the larger SCI population, it still provides important preliminary information for a currently understudied topic. An RCT with a larger sample size is warranted to further examine these findings. Addressing sexual health and sexual function outcomes in future large-scale trials is important to effectively evaluate interventions, achieve best practice standards and improve quality of life for individuals with SCI, especially in the under prioritized population of women with SCI. Our recommendation would be to develop paired intervention strategies that include the addition of cognitive training such as mindfulness, which has been shown to decrease sexual distress in the female neurogenic population (Hocaloski et al., 2016). Future research and treatment of female sexual dysfunction and distress requires a holistic approach involving a multidisciplinary team to address the complexity of sexuality (Elliott et al., 2017).

## Data availability statement

The raw data supporting the conclusions of this article will be made available by the authors, without undue reservation.

## Ethics statement

The studies involving human participants were reviewed and approved by University of Minnesota Institutional Review Board. The patients/participants provided their written informed consent to participate in this study.

## Author contributions

CS: formal analysis, data curation, validation, writing—original draft, visualization. SS: formal analysis, data curation, validation, writing—review and editing. TM: formal analysis, writing—review and editing. AP, US, and TN: conceptualization, methodology, validation, investigation. SH and RS: methodology, validation, writing—review and editing. SE: methodology, validation, writing—review and editing, supervision. MW: methodology, data curation, validation, writing—review and editing, supervision. DD: conceptualization, methodology, resources, validation, investigation, writing—review and editing, supervision, funding acquisition. AK: conceptualization, methodology, investigation, resources, validation, writing—review and editing, supervision. All authors contributed to the article and approved the submitted version.

## Funding

This work was supported by the Minnesota Office of Higher Education, and the University of Minnesota MNDrive. Krassioukov holds Endowed Chair in rehabilitation medicine, University of British Columbia, and his laboratory is supported by funds from the Canadian Institute for Health Research (#PJT-156033), Canadian Foundation for Innovation and BC Knowledge Development Fund (#35869), and PRAXIS Spinal Cord Institute. Dr. Shackleton is supported by a Paralyzed Veterans of America Fellowship (#3189) and the Rick Hansen Foundation (#2007-21). Dr. Samejima is supported by a Paralyzed Veterans of America Fellowship (#3190), Wings for Life Spinal Cord Research Foundation (#2020_097), and the Rick Hansen Foundation (#2007-21). Dr. Miller is supported by the Michael Smith Foundation for Health Research and the Rick Hansen Foundation (#2007-21). Dr. Walter was supported by the Michael Smith Foundation for Health Research and the Rick Hansen Foundation (#17110). Dr. Sachdeva is supported by Wings for Life Spinal Cord Research Foundation (#WFL-CA-20/21).

## Conflict of interest

The authors declare that the research was conducted in the absence of any commercial or financial relationships that could be construed as a potential conflict of interest.

## Publisher’s note

All claims expressed in this article are solely those of the authors and do not necessarily represent those of their affiliated organizations, or those of the publisher, the editors and the reviewers. Any product that may be evaluated in this article, or claim that may be made by its manufacturer, is not guaranteed or endorsed by the publisher.
