# Supplementary material for: Effect of epidural spinal cord stimulation on female sexual function after spinal cord injury
Source: Front Neurosci. 2023 Apr 5;17:1155796. doi: 10.3389/fnins.2023.1155796 (PMC10167769; doi:10.3389/fnins.2023.1155796)
Supplement: Supplementary file 1 [file Data_Sheet_1.docx]

***Supplemental Data***

## **eTable 1. Participant sexual function and distress scores at baseline and after 13 months of epidural spinal cord stimulation**

| **Participant** | **Total** | | **Desire** | | **Arousal** | | **Lubrication** | | **Orgasm** | | **Satisfaction** | | **Pain** | | **Distress** | |
| --- | --- | --- | --- | --- | --- | --- | --- | --- | --- | --- | --- | --- | --- | --- | --- | --- |
|  | *Pre* | *Post* | *Pre* | *Post* | *Pre* | *Post* | *Pre* | *Post* | *Pre* | *Post* | *Pre* | *Post* | *Pre* | *Post* | *Pre* | *Post* |
| 01 | 20.9 | 20.5 | 3.6 | 3.6 | 2.7 | 3.3 | 4.2 | 4.8 | 1.2 | 0.4 | 3.2 | 2.4 | 6.0 | 6.0 | 37.0 | 22.0 |
| 02 | 28.9 | 33.5 | 3.6 | 6.0 | 4.5 | 6.0 | 4.8 | 3.9 | 4.8 | 6.0 | 5.2 | 5.6 | 6.0 | 6.0 | 3.0 | 2.0 |
| 03 | 23.8 | 29.3 | 4.2 | 3.6 | 2.4 | 4.5 | 6.0 | 6.0 | 1.2 | 4.4 | 4.0 | 4.8 | 6.0 | 6.0 | 25.0 | 5.0 |

## **eTable 2: Participant sexual function scores for each month of epidural spinal cord stimulation**

|  | **Female Sexual Function Index Monthly Score (0-13 months)** | | | | | | | | | | | | | |
| --- | --- | --- | --- | --- | --- | --- | --- | --- | --- | --- | --- | --- | --- | --- |
| **Participants** | *0* | *1* | *2* | *3* | *4* | *5* | *6* | *7* | *8* | *9* | *10* | *11* | *12* | *13* |
| 01 | 20.9 | - | - | - | 24.6 | 23.9 | 18.4 | 24.4 | 24.2 | 23.8 | 23.8 | 22.3 | 21.8 | 20.5 |
| 02 | 28.9 | 32.7 | 32.1 | 33.0 | 32.9 | 34.5 | 33.8 | 32.8 | 35.4 | 34.4 | 34.4 | 34.4 | 33.4 | 33.5 |
| 03 | 23.8 | 24.0 | 24.5 | - | 25.9 | 28.5 | 27.9 | 25.3 | 25.6 | 30.6 | 27.8 | 27.1 | 29.2 | 29.3 |

## **eTable 3. Participant medication list at baseline**

| Participant | Medication Name |
| --- | --- |
| 01 | Aspirin  Oxybutynin ER |
| 02 | Baclofen  Gabapentin  Mirena  Xarelto  Zoloft  Florinef  Epinephrine  Bactrim  Macrobid |
| 03 | None |
